# Flat irregular pigment epithelial detachment over time and outcome of different treatment regimens

**DOI:** 10.1038/s41598-022-14762-1

**Published:** 2022-06-24

**Authors:** Nazanin Ebrahimiadib, Mohammadreza Mehrabi Bahar, Hamid Riazi-esfahani, Elias Khalili Pour, Fariba Ghassemi, Hooshang Faghihi, Ahmad Mirshahi, Ramak Roohipourmoallai, Alireza Lashay, Alireza Mahmoudi, Kaveh Fadakar

**Affiliations:** 1 Eye Research Center, Farabi Eye Hospital, Retina Services, Tehran University of Medical Sciences, South Kargar Street, Qazvin SquareTehran, 1336616351 Iran; 2 Department of Ophthalmology, Morsani College of Medicine, University of South Florida, Tampa, FL USA

**Keywords:** Diseases, Medical research

## Abstract

To present long-term visual and structural outcome of treatment in two forms of flat irregular pigment epithelial detachment (FIPED): avascular (aFIPED) and vascularized (vFIPED) in eyes within pachychoroid spectrum. Prospective interventional case series. FIPED were classified into two subgroups; aFIPED and vFIPED based on OCTA. aFIPED underwent PDT, and vFIPED underwent either PDT, IVB, or combination of PDT&IVB. Vision, subretinal or intraretinal fluid, and choroidal biomarkers such as choroidal thickness, area, choroidal vascular index (CVI), and PED area were measured at baseline and last follow-up. Fifteen eyes with aFIPED were followed for a mean of 14.7 ± 10.8 months. Their vision improved, (0.44 ± 0.37–0.33 ± 0.40 LogMAR, *p* = 0.009) with significant reduction of fluid, choroidal area, thickness, PED area and increase in CVI. Twenty eyes with vFIPED were followed for a mean of 16.5 ± 8.2 months. The same pattern of choroidal alterations without visual improvement was observed in eyes underwent PDT alone. Combination therapy resulted in improvement of vision (0.38 ± 0.10–0.23 ± 0.17 LogMAR, *p* = 0.006) with reduction of choroidal area and thickness, with an increase in CVI. IVB alone could not change vision or choroidal structure. Single session PDT may lead to sustained visual improvement and structural change in eyes with aFIPED. Combination of PDT and IVB may be a better choice in eyes with vFIPED.

## Introduction

The hallmark of pachychoroid spectrum is choroidal vascular dilation in the Haller’s layer (pachyvessels), which results in thinning of the choriocapillaris and Sattler’s layer. The earliest stage of complicated pachychoroid begins with focal changes in retinal pigment epithelium (RPE) and formation of pigment epithelial detachment (PED), which is stimulated by the underlying choroidal vascular hyperpermeability and congestion^1^. These RPE abnormalities are usually located above the pachyvessels, which may later progress to central serous chorioretinopathy (CSC) and finally to pachychoroid neovasculopathy (PNV)^1,2^.

PED happens in 70–100% of eyes with CSC and usually appears as a dome-shaped serous PED in acute CSC and as a flat irregular pigment epithelial detachment (FIPED) in chronic CSC^3^. Previous studies reported that 30 to 35% of FIPED may harbor type I neovascular complex^3–6^, which implies the diagnosis of PNV. Therefore, in a narrow segment in the pachychoroid spectrum we have two categories of avascular FIPED (in the setting of a chronic CSC) and vascularized FIPED (transformation of a chronic CSC to PNV) in the proximity of each other. It is known that with increasing ischemic impact induced by dilated vessels in choroidal layer, neovascularization occurs with increasing vascular endothelial growth factor (VEGF) levels. Transformation of aFIPED to the form of vFIPED mandates treatment strategies to combat strongly with this increasing levels of VEGF. However, there is no standard guidelines set for the treatment of these entities. In the present study, we followed eyes with FIPED (vascular and avascular) and serous retinal detachment for a relatively long-term and investigated their clinical course, response to treatment and alterations in PED area, and choroidal biomarkers in each category of aFIPED and vFIPED separately.

## Materials and methods

### Study population

This was a prospective interventional cohort study conducted in a tertiary eye center, Farabi hospital. Patients with the diagnosis of FIPED and serous retinal detachment who were referred to the retina clinic, from March 2019 to September 2020, were included.

The study protocol and procedures adhered to the principles outlined in the Declaration of Helsinki and were approved by the Tehran University of Medical science’s Institutional Review Board. Informed consent was obtained from each participant before inclusion in the study.

Patients with thick choroid/dilated vessels in Haller/Sattler layer, attenuated choriocapillaris and abnormalities in RPE were considered for this study. All patients included should harbor subretinal fluid. The diagnosis of macular FIPEDs was made based on the presence of an irregularly contoured separation of the RPE from Bruch’s membrane (BM) on enhanced-depth imaging optical coherence tomography (EDI-OCT) cross-sectional B-scans. Then all FIPEDs were classified as aFIPED or vFIPEDs based on the presence of vascular tuft according to the evaluation by OCTA, enface images and corresponding flow overlay B-scans. Eyes showing characteristics of polypoidal choroidal vasculopathy in OCTA/OCT were excluded from the study^7,8^.

Patients with decreased visual auity were enrolled for treatment. Those patients with aFIPED received half dose PDT and those with vFIPED, were allocated to one of the three treatment subgroups mentioned below. They follow up clinical and imaging data of those who completed at least 6 months follow up were used for analysis.

Treatment protocols: Patients with aFIPED were treated with half-dose PDT, and eyes with vFIPED were non-randomly, and based on accessibility assigned to one of treatment modalities; half-dose PDT, intravitreal anti-VEGF injection, or a combination of both. Half-dose PDT was performed with half dose verteporfin which was administered over 10 min. Five minutes after termination of the infusion, a 689-nm laser was applied to the pathologic area for 83 s to deliver an overall dose of 50 J/cm^2^. The area to cover was determined based on the greatest linear dimension of choroidal hyperpermeability demonstrated by indocyanine green angiography (ICGA). Treatments were performed by one of two physicians; NE and HRE.

For those with vFIPED who were assigned to anti-VEGF injection, three monthly consecutive injections of intravitreal bevacizumab (IVB) were performed. In combination therapy, three monthly injections were scheduled for the patients and half-dose PDT was performed within two weeks from the first injection,. In both subgroups, further pro re nata injections were administered if the intraretinal fluid (IRF) persisted.

Patients below 18 or above 80 years-old, refractive error (spherical equivalent) ≥ ± 3 diopters, current or prior retinal pathologies such as any sign of age-related macular degeneration (ARMD), retinal detachment or retinal vascular lesions, those with poor EDI-OCT image quality, history of cataract surgery within the past 6 months, history of any other major ocular surgery, prior photodynamic therapy (PDT) or laser photocoagulation, intravitreal anti-VEGF injection, glaucoma, optic nerve abnormalities and intraocular inflammation were excluded. Patients with systemic conditions that may affect hemodynamic regulation such as systemic hypertension (systolic BP > 150 mm Hg or diastolic BP > 90 mm Hg) or diabetes were also excluded from the study.

Patients’ demographic data, type of treatment applied and the duration of follow up time after treatment were recorded. A full ophthalmological examination including best-corrected visual acuity (BCVA) based on Snellen chart, intraocular pressure and fundus examination, as well as EDI-OCT (RTVue XR 100 Avanti instrument Optovue, Inc., Fremont, CA, USA) and optical coherence tomography angiography (OCTA) (AngioVue, Optovue, Inc., Fremont, CA, USA) were performed for all patients at baseline and during follow up. Optometrists who measured BCVA and operators of imaging devices were masked to diagnosis and treatment group. Also, in every visit, the presence of SRF and IRF involving the fovea were recorded. For the sake of the study, if there was no unexpected event, we considered the last follow up image for analysis. All choroidal parameters including Subfoveal choroidal thickness (SFCT), total choroidal area (TCA), and CVI, as well as PED area were measured at baseline and follow up visit. For all patients who were assigned to PDT, ICGA (Heidelberg Spectralis, Heidelberg Engineering, Germany) was performed. Fluorescein angiography (FA) (Heidelberg Spectralis, Heidelberg Engineering, Germany) was an additional imaging requested for those categorizing of whom to aFIPED or vFIPED could not be discerned satisfactorily based on OCT and OCTA.

### Image acquisition and analysis protocol

**Figure 1:**
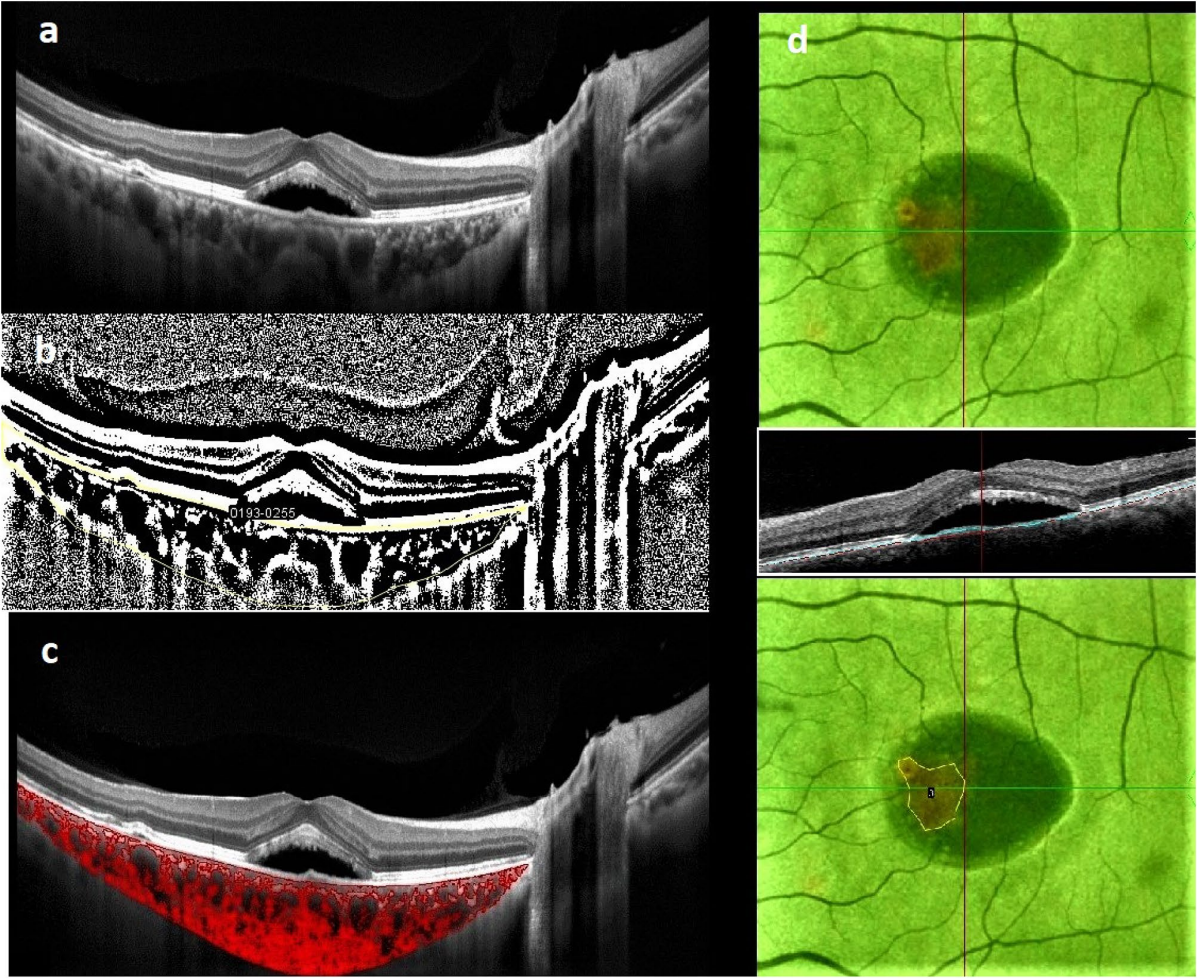
Measurement of choroidal parameters: subfoveal choroidal thickness is measured manually from the base of RPE to the innermost border of choroidoscleral junction (**a**). Choroidal area is delineated followed by binarization of the region of interest (**b**). The image is then converted to RGB using color threshold, and the choroidal vascular index is calculated by dividing areas without pixels as choroidal lumens to total choroidal area (**c**). PED area is evident in en-face RPE map as hot regions, which is manually delineated (**d**).

Patients were positioned appropriately and equally spaced OCT B-scans at 8 mm × 12 mm raster patterns were captured. The scan passing through the fovea was selected for image analysis. Low-quality scans in which choroidoscleral junction was not discernible were omitted. SFCT, defined as the distance between the outermost border of BM-RPE complex and the innermost border of choroidoscleral junction in the subfoveal region, was measured manually with the aid of built‑in calipers in OCT software by two masked observers (AM, RMB) (Fig. 1a). In case of any dispute, the outlines were segmented by consensus of other authors (NE, HR, KF). All EDI-OCT scans were performed between 8:00 and 11:00 to overcome diurnal variations. Then OCTA was performed to investigate the outer retina and choriocapillaris slab for the presence of macular neovascularization.

### Measurement of total choroidal area and CVI by binarization technique

Discerned independently by unmask graders, good quality EDI-OCT images were selected to be reviewed. Image analysis was conducted by FIJI (an expanded version of ImageJ software, version 1.51 h; National Institutes of Health, Bethesda, Maryland, available at http://imagej.nih. gov/Fiji/). The borders of choroid were selected using free hand tool of the software. The upper margin was RPE and the lower margin was the choroidoscleral border. The nasal margin was the temporal edge of the optic nerve head and the temporal margin was 8 mm from the temporal edge of the optic nerve head. To binarize choroidal area in OCT images, a modified Niblack method was used as previously described^9^. Briefly, three choroidal vessels with lumens larger than 100 µm were randomly selected by the oval selection tool of the toolbar, and the average reflectivity of these areas was determined by the software. The average brightness was set as the minimum value to reduce the noise in the OCT image. The ROI was selected and set by the ROI manager in the OCT image. Then, the image was converted to 8 bits and adjusted by the auto local threshold of Niblack. The binarized image was reconverted to an RGB image, and the luminal area was determined using the color threshold tool. The light pixels were described as the choroidal stroma or interstitial area and the dark pixels were defined as the luminal area (LA). TCA, LA, and stromal area (SA) was automatically calculated. Herein, we refer to the ratio of LA to TCA as the choroidal vascular index (CVI) (Fig. 1b, c).

### Measurement of PED area

En face RPE elevation map were used to depict the total PED area (Fig. 1d). Images were imported to FIJI and scales were set accordingly. Borders of PED, which were demonstrated as hot colors in heat map image provided by the built-in software of the device, were manually selected using the free hand tool of FIJI. PED area at baseline and follow-up images were measured and used for analysis.

### Statistical methods

To describe data, we used mean, standard deviation, median, range, frequency, and percentage. To compare the results between groups when considering the probable correlation of the eyes, we used generalized estimating equation (GEE). Multiple comparisons are considered by the Sidak method. Association of baseline predictive factors to the alteration of BCVA were assessed using linear model within GEE method. To compare intergroup baseline characteristics we performed ANOVA test, and post hoc pairwise comparison was adjusted with Sidak method. Comparison of intergroup follow-up characteristics was performed using ANCOVA, which adjusts possible confounding effect of baseline values. A *P* value of less than 0.05 is considered statistically significant. All statistical analysis was performed by SPSS (IBM Corp. Released 2017. IBM SPSS Statistics for Windows, Version 25.0. Armonk, NY: IBM Corp). KF did the statistical analysis, and was not masked to the labels of group identifiers and treatment subgroups.

## Results

### Baseline characteristics

A total of 35 eyes, fifteen eyes of 11 patients with aFIPED and 20 eyes of 15 patients with vFIPED, were enrolled in this study. Table 1 demonstrates the baseline characteristics of the subjects. In regard to demographic data, choroidal parameters, presence of SRF, IRF and PED area there was no significant difference between the two groups.

**Table 1:** Baseline characteristics of patients with aFIPED and vFIPED.

	Avascular FIPED N = 15	Vascularized FIPED N = 20	*P* value
Age	48.27 ± 12.15	51.55 ± 9.37	0.481
Sex (male)	13 (86.7%)	14 (70%)	0.756
Best corrected visual acuity (logMAR)	0.46 ± 0.36	0.46 ± 0.30	0.921
Subretinal fluid	15 (100%)	18 (90%)	0.319
Intraretinal fluid	0 (0%)	3 (15%)	0.244
Total choroidal area (mm^2^)	1.29 ± 0.36	1.25 ± 0.36	0.587
Subfoveal choroidal thickness (µm)	428 ± 108 (range 308–633)	391 ± 102 (range 266–649)	0.144
Choroidal vascular index	70.09 ± 3.32	70.19 ± 3.19	0.592
PED area (mm^2^)	1.02 ± 1.19	0.98 ± 0.80	0.781

The overall mean follow up time was 15.7 ± 9.1 months; 14.7 ± 10.8 months for aFIPED and 16.5 ± 8.2 months for vFIPED. Diameter of total area treated with PDT was 5700 ± 3329 µm in aFIPED, 6080 ± 3391 µm in vFIPED eyes treated with PDT, and 6000 ± 4359 µm in vFIPED eyes in combination treatment subgroup. Statistical analysis revealed no significant difference regarding PDT area among groups (*p* value: 0.978).

**Figure 2:**
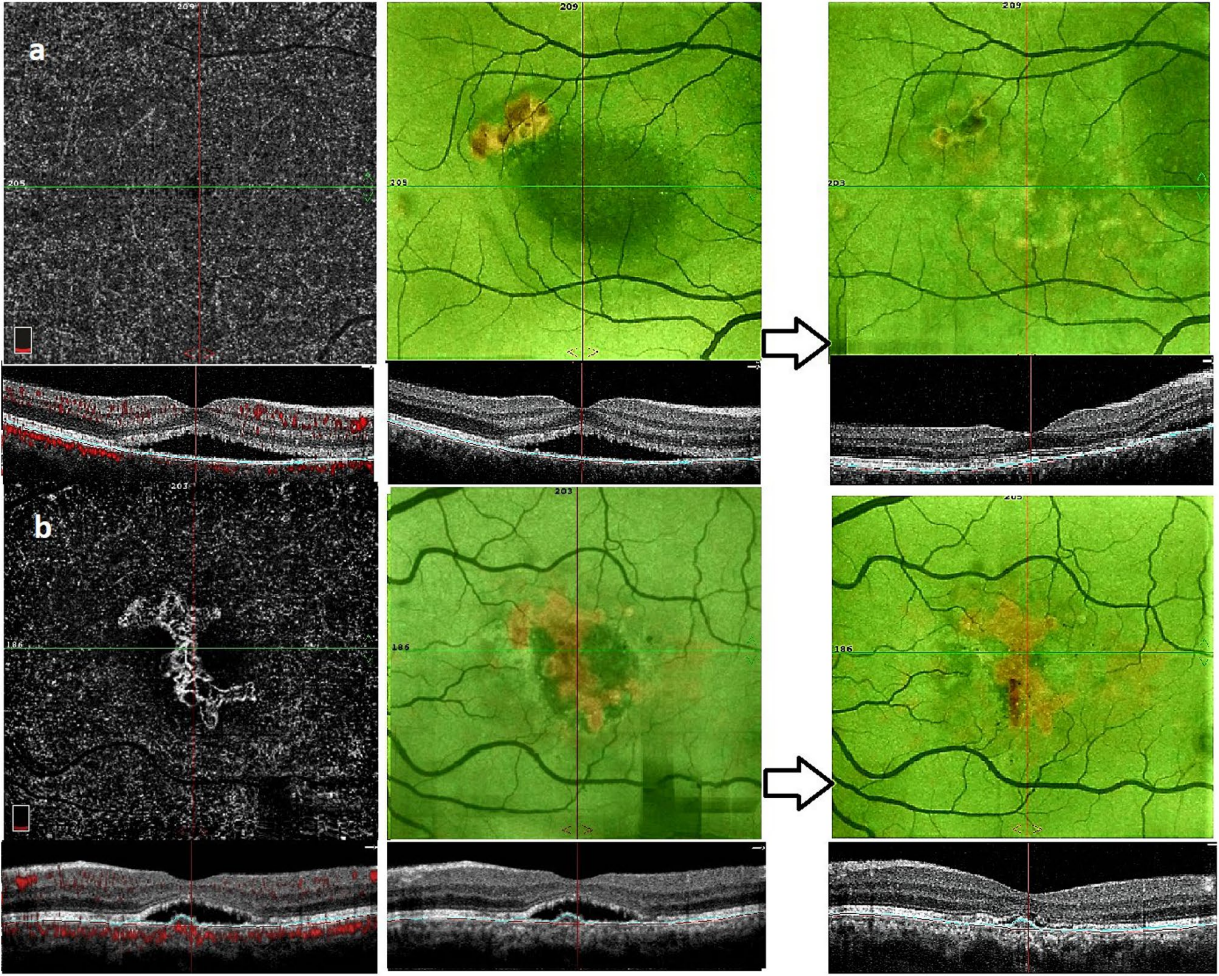
Representative baseline and follow up of eyes with aFIPED (**a**) and vFIPED (**b**): OCT, OCTA slab, and en-face RPE map depicts subretinal fluid (SRF) without vascular network and hot area of shallow irregular PED. Following PDT, subretinal fluid has resolved accompanied by reduction in PED area (**a**). OCTA in vFIPED clearly demonstrates the vascular network (**b**). SRF is evident in OCT and borders of PED area illustrated in en-face RPE map. Following combination therapy with PDT and three consecutive IVB injection, SRF is resolved with improvement of vision; however PED area remained relatively unchanged.

Figure 2 is representative of treatment with PDT in an eye with aFIPED, and combination therapy in an eye with vFIPED.

### Avascular FIPED

Fourteen eyes with aFIPED underwent half dose PDT. One patient refused treatment. His BCVA was 0.7 logMAR at baseline, which reduced to 1 logMAR after 12-month follow-up. At the last follow-up visit (mean: 15.7 months) of those who received treatment, a significant improvement in vision was observed (*p* = 0.009). SRF in the absence of IRF were present in all eyes which resolved in 9 (60%) eyes (*p* = 0.005). In eyes with persistent SRF, final BCVA was 0.30 ± 0.35 logMAR which was not different from final BCVA in eyes with complete resolution of SRF, 0.34 ± 0.45 logMAR (*p* = 0.882). Following treatment, PED area, choroidal thickness and TCA decreased significantly, while CVI increased significantly (Table 2 and Fig. 2a).

**Table 2:** Alteration of vision, macular fluid status, choroidal parameters and PED area after half dose photodynamic therapy in patients with avascular FIPED.

	Baseline	After treatment	*P* value
Best corrected visual acuity (logMAR)	0.44 ± 0.37	0.33 ± 0.40	**0.009**
Intra retinal fluid	0 of 14	0 of 14	–
Subretinal fluid	14 of 14 (100%)	5 of 14 (35%)	**< 0.001**
Total choroidal Area (mm^2^)	1.29 ± 0.36	1.16 ± 0.34	**< 0.001**
Subfoveal choroidal thickness (µm)	422 ± 106	395 ± 112	**< 0.001**
Choroidal vascular index	69.9 ± 3.32	72.88 ± 3.41	**0.023**
PED area (mm^2^)	1.02 ± 1.19	0.59 ± 1.08	**0.016**

### Vascularized FIPED

From twenty eyes with vFIPED, two eyes from two patients did not receive any treatment due to good baseline visual acuity (log MAR = 0.00 in both). They maintained this vision until the last follow-up visit (8 months in one patient and 30 months for the other one). SRF was present in all eyes, while IRF was observed in three eyes in this category. Nine eyes were treated with half dose PDT, five received IVB injections (number of injections; median: 6, range 4–11), and four eyes underwent combination therapy with both PDT and three monthly IVB injections (Table 3). There were not any significant differences in baseline values between the three treatment subgroups in vFIPED. At the final follow-up visit, eyes that underwent combination therapy compared to IVB group, showed an improvement in visual acuity (0.23 ± 0.17 vs 0.54 ± 0.21, *p* value: 0.006) and choroidal area (0.86 ± 0.19 vs 1.16 ± 0.25, *p* value: 0.028). Final visual acuity and choroidal parameters were not different between the other treatment subgroups.

**Table 3:** Alteration of vision, macular fluid status, choroidal parameters, and PED area after different treatment modalities in eyes with vFIPED.

	PDT (n = 9)	IVB (N = 5)	Combination (N = 4)	Intergroup analysis *P* value
				Overall
**BCVA (logMAR)**				
Baseline	0.60 ± 0.32	0.45 ± 0.24	0.38 ± 0.10	0.347
Treated	0.56 ± 0.32	0.54 ± 0.21	0.23 ± 0.17	**0.006**
*Within group P value*	0.118	0.075	**0.006**	
**Intraretinal fluid**				
Baseline	1 of 9 (11.1%)	0 of 5 (0%)	2 of 4 (50%)	0.111
Treated	1 of 9 (11.1%)	1 of 5 (20%)	0 of 4 (0%)	0.638
*Within group P value*	0.999	0.292	0.102	
**Subretinal fluid**				
Baseline	9 of 9 (100%)	5 of 5 (100%)	4 of 4 (100%)	–
Treated	5 of 9 (55.5%)	5 of 5 (100%)	2 of 4 (50%)	0.174
*Within group P value*	**0.023**	–	0.102	
**TCA (mm**^**2**^**)**				
Baseline	1.34 ± 0.40	1.44 ± 0.30	1.01 ± 0.14	0.260
Treated	1.16 ± 0.25	1.46 ± 0.13	0.86 ± 0.19	**0.029**
*Within group P value*	**0.010**	0.865	** < 0.001**	
**SCT (µm)**				
Baseline	434 ± 121	379 ± 54	353 ± 56	0.396
Treated	398 ± 87	352 ± 85	306 ± 68	0468
*Within group P value*	**0.036**	0.087	**0.003**	
**CVI**				
Baseline	69.8 ± 2.97	69.8 ± 4.91	70.5 ± 3.91	0.944
Treated	72.2 ± 2.52	69.1 ± 4.60	73.5 ± 3.37	0.086
*Within group P value*	**0.002**	0.901	** < 0.001**	
**PED area (mm**^**2**^**)**				
Baseline	0.760 ± 0.776	NA	1.162 ± 0.796	0.322
Treated	0.411 ± 0.477	NA	1.153 ± 0.912	0.129
*Within group P value*	0.235	–	0.967	

In those who received half dose PDT, at last mean follow up time of 13.6 ± 5.3 months, a non-significant improvement in BCVA was observed. SRF resolved in 4 of 9 (45.5%) eyes (*p* value = 0.023) and IRF which was observed in one eye at baseline persisted despite treatment. The reduction in PED area was not statistically significant. However, a significant reduction in choroidal area and subfoveal choroidal thickness was observed, while CVI increased substantially at the last follow-up visit.

Five eyes received IVB injections and were followed for an average of 15.8 ± 6.4 months, a not-significant worsening in BCVA was observed (*p* value = 0.075). SRF persisted in all eyes, and IRF developed in one patient after the conclusion of treatment. Choroidal area, thickness, and CVI did not change significantly. Measurement of the PED area was not possible due to poor baseline image quality.

Four eyes received the combination of PDT and 3 injections of IVB, and were followed for an average of 22.75 ± 10.9 months. Following treatment, BCVA improved significantly. SRF resorbed in half of the eyes and IRF which was observed in 2 eyes at baseline, completely resolved with combination treatment. Decrease in choroidal area and thickness, as well as increase in CVI was significant. PED area remained unchanged (Fig. 1b).

In vFIPED, excluding eyes received IVB only treatment, final BCVA was similar in eyes with residual and resolved SRF (0.44 ± 0.41 vs. 0.47 ± 0.20 logMAR, *p* = 0.896).

Association of baseline characteristics and choroidal parameters with vision improvement:

Association of age, sex, baseline TCA, SFCT, CVI, and PED area was investigated with alteration of BCVA after treatment in patients with aFIPED and vFIPED (Table 4). In eyes with aFIPED, baseline PED area and CVI was associated with vision improvement. In eyes with vFIPED, age showed to have negative association with vision improvement.

**Table 4:** Association of baseline characteristics with alteration of vision following treatment in eyes with aFIPED and vFIPED.

	Variables	Baseline values	B (confidence interval 95%)	*P* value
aFIPED N = 14 Alteration of BCVA: − 0.07 ± 0.14	Age (years)	48.27 ± 12.15	− 0.005 (− 0.01 to 0.001)	0.064
	Sex (male)	12 (86.7%)	0.042 (− 0.047 to 0.132)	0.353
	Total choroidal area (mm^2^)	1.29 ± 0.36	0.311 (− 0.150 to 0.773)	0.186
	Subfoveal choroidal thickness (µm)	422 ± 106	0.01 (− 0.002 to 0.001)	0.612
	CVI	69.9 ± 3.32	− 0.045 (− 0.052 to − 0.039)	** < 0.001**
	PED area (mm^2^)	1.02 ± 1.19	− 0.093 (− 0.133 to − 0.052)	** < 0.001**
vFIPED N = 18 Alteration of BCVA: − 0.02 ± 012	Age (years)	51.28 ± 9.86	0.009 (0.00–0.011)	**0.032**
	Sex (male)	13 (72.2%)	− 0.177 (− 0.234 to 0.120)	0.482
	Total choroidal area (mm^2^)	1.26 ± 0.36	0.062 (− 0.007 to 0.131)	0.077
	Subfoveal Choroidal thickness (µm)	403 ± 101	0.000 (0.000–0.000)	0.683
	CVI	69.98 ± 3.18	0.010 (− 0.002 to 0.022)	0.103
	PED area (mm^2^)	0.98 ± 0.80	− 0.018 (− 0.075 to 0.039)	0.532

## Discussion

We prospectively evaluated treatment response in eyes with subretinal fluid and FIPED in the spectrum of pachychoroid disease. We categorized FIPED into two groups based on OCTA findings; avascular and vascularized. We showed that in eyes with aFIPED, half-dose PDT could effectively induce an improvement in visual acuity, reduction in PED area, as well as choroidal area and thickness. In eyes with vFIPED, a better result for vision improvement was observed in the subgroup received combination therapy with half-dose PDT and IVB injections. To our knowledge, this study is the first investigation that compares treatment response in these two adjacent entities reporting visual, clinical, and structural outcome after a relatively long term follow up.

Single session appropriate treatment in both aFIPED and vFIPED could lead to a durable vision improvement and structural remodeling in a relatively long term follow up of approximately 16 months. Limitations related to COVID-19 pandemic, and global shortage of verteporfin were obstacles in the current study to have fixed time follow ups or to repeat treatment sessions for eyes with persistent subretinal fluid. Considering this fact, our results might represent an example of real-world practice pattern and fill the gap of knowledge of the long-term outcome of single session PDT in these entities. It is noteworthy that in all categories of our study, with administration of initial treatment, no significant worsening of visual acuity was observed and visual stability or improvement was achieved in more than 88% of eyes.

Earlier reports demonstrated that PDT functions as a promising mode of treatment for chronic CSC which results in reduction of choroidal thickness and hyper-permeability as well as resolution of SRF^10^. Our result confirmed that half-dose PDT, alone or in combination with anti-VEGF, could induce a significant resolution of SRF in more than half of our patients, considering both avascular and vascularized FIPED group. Several studies confirmed that in eyes with choroidal neovascularization, the persistence of a stable SRF per se, is not an indication for retreatment. Therefore, the remaining SRF may not raise a concern in our vFIPED group. In this category, vision of eyes with residual SRF was similar to eyes with complete resolution of SRF with no significant difference. Interestingly, the same pattern was observed in eyes with aFIPED. In our aFIPED group following PDT, the significant improvement in visual acuity maintained until the last follow up visit at months 18, despite the retention of SRF. Of note, we had one patient in aFIPED group who rejected treatment and showed worsening of vision during follow up. This observation may emphasize the effectivity of PDT, however the necessity of retreating the residual SRF with subsequent PDTs may be dependent on other determinants such as findings in ICGA which should be investigated in future larger controlled trials. It is noteworthy that most of studies emphasize on omnious effect of persistent IRF on visual acuity. In our study population, we have only one patient with persistent IRF which happened after PDT. He had a poor baseline visual acuity of 1.3 LogMAR which remained the same in the last follow up visit at 12 months. One patient developed IRF after 11 IVB injections during 22 months and her vision decreased from 0.15 LogMAR at baseline to 0.4 LogMAR at final follow up visit.

Our observation regarding the better treatment outcome achieved with combination therapy, indicates that in eyes with PNV, abnormality of the choroid and neovascular component should be addressed together. The current theory, explaining the development of PNV in pachychoroid disease spectrum, suggests that enlarged choroidal vessels compress and attenuate the choriocapillaris which may induce ischemia and subsequent neovascularization response^11,12^. However, the development of SRF may originate from both CNV activity and exudation of choroidal vessels. Therefore, an effective therapy should tackle both pathologies and, in this regard, combination therapy seems to be a reasonable approach. Several studies reported favorable results with combination of PDT and aflibercept or with PDT as an adjuvant therapy after unresponsiveness to anti-VEGF^13,14^. Our study confirms that eyes with vFIPED demonstrate a promising and durable outcome with combination therapy of PDT and bevacizumab. Although changes in vision is significant within combination therapy subgroup, further analysis between three treatment subgroups showed that despite higher trend in combination treatment subgroup, final vision does not statistically differ between PDT and combination therapy in eyes with vFIPED. This may imply that the PDT and combination therapy are both effective to stabilize or improve vision in vFIPED. Although our sample size is small to draw a robust conclusion in this regard.

We did not detect any effect on vision, macular fluid and choroidal parameters in the bevacizumab group. Controversy remains regarding the effect of anti-VEGF for the treatment of vFIPED. Although some studies showed favorable outcome with anti-VEGF therapy, such observations were not reproduced in other studies. Pardon-perez et al. treated 18 PNV patients with anti-VEGF and reported a reduction in subfoveal choroidal thickness without significant visual gain^15^. The controversy may be related to different efficacy of various anti-VEGF agents, and probable superiority of aflibercept to other agents^16,17^, or different response of type I CNV in eyes with PNV from those with neovascular AMD (nAMD). Aqueous level of VEGF is significantly lower in patients with PNV in comparison to nAMD^18,19^ and the chances of persistent fluid after anti-VEGF treatment is higher in eyes with PNV than nAMD. These findings imply that the activity of PNV may be modulated by factors other than VEGF.

It is postulated that the modified PDT may spare choriocapillaris and induce remodeling of large caliber vessels which leads to reduction of the luminal area without concomitant choriocapillaris hypoperfusion^20,21^. In parallel with shrinkage of dilated vessels, the stromal area will also decrease. This is reflected in our result, as reduction of choroidal area is accompanied by an increase in CVI, indicating that reduction of the congestion of interstitial tissue is a consequence of effective treatment. PED area is also significantly reduced following treatment in eyes with aFIPED. Baseline value of both CVI and PED area are associated with visual gain in eyes with aFIPED. However, in eyes with vFIPED, even after successful treatment with complete resolution of fluid, PED area remains unchanged. Higher reflectivity within PED in eyes with vFIPED, compared to aFIPED, indicate its vascular content^22^. Previous studies showed that vessel density and explant area do not change after successful treatment of PNV^23^. Therefore, PED diameters as well as area do not alter significantly after successful management of this group of patients. This finding was confirmed in our series.

There are several limitations for our study. First, it is a non-randomized study with two physicians treating patients. Our statistician was not masked as well. These confounding factors make our results subject to bias. Also, we lack ETDRS chart for BCVA measurement. Additionally, we have a small sample size and the number of cases in each group is not equal. Although, we tried to compensate for it with precise statistical methodology, further prospective controlled studies are required to validate our results. Lack of a control group is another limitation of our study. Having a control group could provide data regarding the natural course of the disease and confirm the effect of various treatment modalities. Also, the duration of symptoms was not specified which might affect the treatment outcome. Another limitation of the current study was that we did not evaluate all of our patients with dye-based angiographies (FFA/ ICGA) which could be helpful to investigate for the polypoidal lesions; however recent studies compared OCTA and ICGA for detection of polypoidal lesion and reported a high accuracy for OCTA^7^. We should keep in mind that for entities with such insidious course, longer term follow up is required to determine the sustainability of treatment and the need for repeat treatment in case of recurrences.

In summary, in eyes with aFIPED, single session half dose PDT provided a favorable visual outcome accompanied by SRF resolution, and reduction of choroidal thickness and PED area. In eyes with vFIPED, combination therapy with single session half dose PDT and multiple anti-VEGF injections gave a promising outcome with improvement in vision, macular fluid and choroidal parameter. Only one session half dose PDT could still help stabilize vision, resolve the intraretinal and subretinal fluid and improve the choroidal parameters in eyes with vFIPED. Assessment of the standard treatment protocol in patients with pachychoroid spectrum and whether they can maintain visual acuity with lower treatment burden requires further larger studies. Overall, Randomized-controlled trials are necessary to substantiate our findings in this study.

## Data Availability

Derived data supporting the findings of this study including images, and statistical data set is available from the corresponding author on request.
